# Two-stage hepatectomy aiming for the development of intrahepatic venous collaterals for multiple colorectal liver metastases

**DOI:** 10.1186/s40792-018-0424-5

**Published:** 2018-02-16

**Authors:** Harufumi Maki, Shouichi Satou, Kentaro Nakajima, Atsuki Nagao, Kazuteru Watanabe, Hitoshi Satodate, Satoshi Nara, Kaoru Furushima, Yasushi Harihara

**Affiliations:** Department of Surgery, NTT Medical Center Tokyo, 5-9-22 Higashi-Gotanda, Shinagawa-Ku, Tokyo, 141-8625 Japan

**Keywords:** Two-stage hepatectomy, Intrahepatic collateral, Colorectal liver metastasis, Postoperative liver failure, Liver congestion

## Abstract

**Background:**

Aggressive hepatectomy with venous resection has a higher risk of postoperative liver failure (POLF) than hepatectomy without venous reconstruction; however, venous reconstruction is technically demanding. We performed a novel two-stage hepatectomy (TSH) without venous reconstruction in a patient with bilobar multiple colorectal liver metastases located near the caval confluence, waiting for the development of intrahepatic venous collaterals between procedures.

**Case presentation:**

A 60-year-old man was referred to our hospital with sigmoid colon cancer accompanied by intraabdominal abscess and two synchronous liver metastases. One of the liver tumors (tumor 1) was located in segment 8 near the caval confluence and was attached to both the right hepatic vein (RHV) and middle hepatic vein (MHV). The other tumor (tumor 2) in the left lobe invaded the umbilical portion of the portal vein. Both liver metastases decreased in size after four cycles of panitumumab/5-fluorouracil, leucovorin, and oxaliplatin (FOLFOX) therapy. Radical liver resection was planned because tumor 1 had not invaded the MHV. However, three-dimensional volumetric software showed that the non-congested volume of the future liver remnant was estimated at 354 ml, which corresponded to 26.3% of the total liver volume. TSH was scheduled to avoid POLF. We first performed limited resection of segment 8 with resection of the RHV root. After the first hepatectomy, the development of intrahepatic venous collaterals between the RHV and MHV was seen on computed tomography and magnetic resonance imaging. The estimated non-congested future liver remnant was 1242 ml, 78.5% of the total liver volume. Therefore, the patient underwent left hemihepatectomy 58 days after the first hepatectomy. We saw no adhesions around the porta hepatis, and the left hepatic artery and left branch of the portal vein were safely exposed and divided. Intraoperative Doppler ultrasonography revealed intrahepatic venous collaterals arising from RHV to MHV. The patient’s postoperative course was uneventful, and he underwent eight cycles of panitumumab/FOLFOX therapy for 5 months after the second hepatectomy.

**Conclusions:**

Our TSH strategy helped avoid POLF by waiting for the development of intrahepatic venous collaterals.

## Background

Liver resection is the only potentially curative treatment for colorectal liver metastases [1]. However, extensive resection risks postoperative liver failure (POLF) and high mortality. Before operating, we must confirm a sufficient future liver remnant (FLR) to maintain adequate vascular inflow/outflow after hepatectomy for bilobar multiple tumors. Liver resection is technically demanding when the tumor is located near the caval confluence because of its deep location and possible invasion to the major hepatic veins [2]. In such cases, major hepatectomy with venous resection may increase the risk of POLF not only because the FLR volume is small but also because liver function is impaired in the congested area as a result of severing the major hepatic veins [3, 4]. Reconstructing the hepatic veins avoids liver congestion [3, 5–9]; however, reconstruction is not easy in all cases.

Two-stage hepatectomy (TSH) is a feasible procedure for multiple liver tumors that are considered unresectable or that have a high risk of POLF following one-stage hepatectomy. Oncological outcomes and postoperative complications of TSH are favorable compared with one-stage procedures [1, 10]. Therefore, we discuss a new TSH technique for multiple colorectal metastases near the caval confluence that waits for the development of intrahepatic venous collaterals to avoid POLF. In the patient in this case report, reconstruction of the hepatic veins was not required because hepatic venous flow was compensated by intrahepatic venous collateral development [11].

## Case presentation

**Fig. 1:**
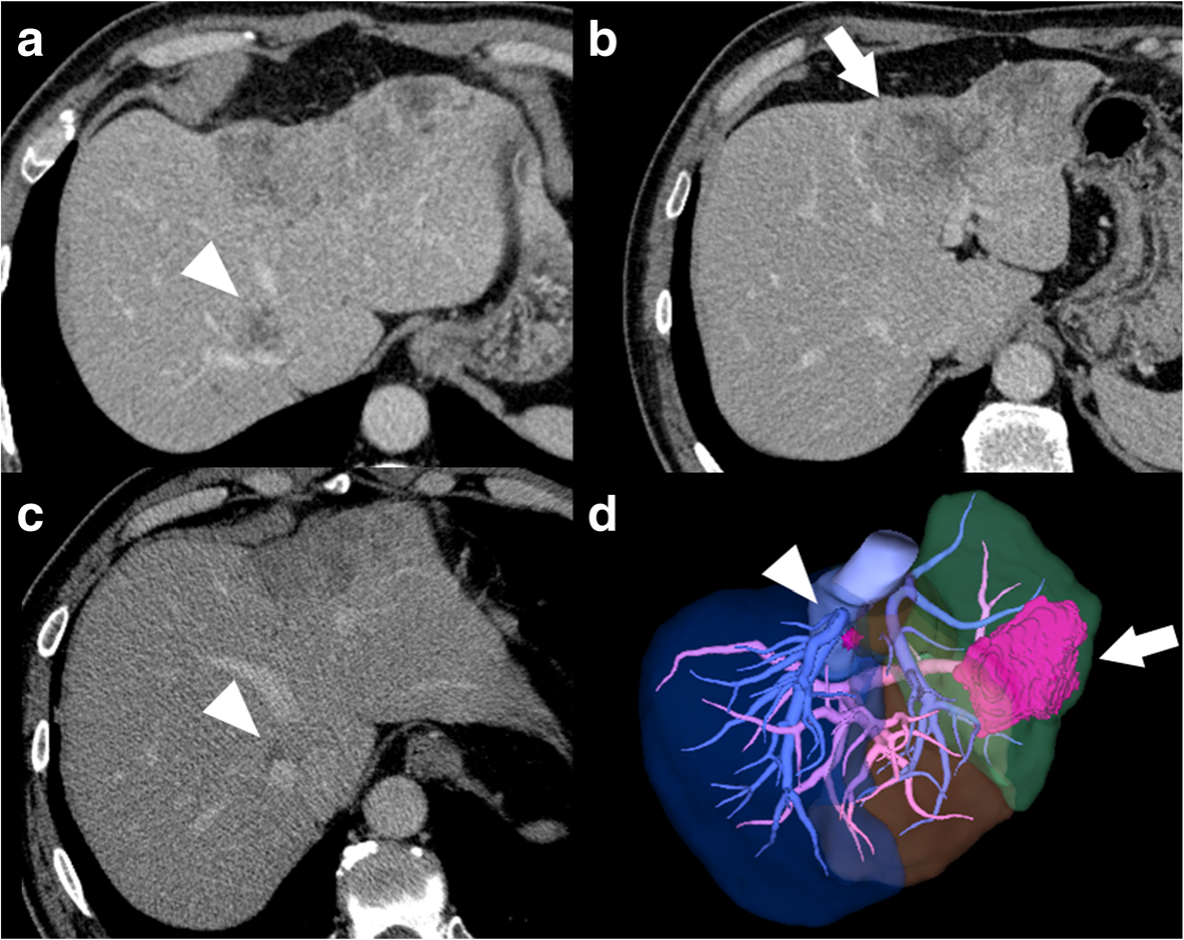
Preoperative images during the first hepatectomy. **a** Tumor 1 was located in segment 8 near the caval confluence (*white arrowhead*). The borders between the tumor and the right or the middle hepatic veins were unclear. **b** Tumor 2 was located in the left lobe and had invaded the umbilical portion of the portal vein (*white arrow*). **c** Tumor 1 decreased in size after chemotherapy but was still attached to the RHV (*white arrowhead*). **d** Tumor 1 (*white arrowhead*) and tumor 2 (*white arrow*) were visualized by three-dimensional volumetric software. The volumes of the left liver, the non-congested area, and the congested area were estimated at 19.6% (*green-colored area*), 26.3% (*brown-colored area*), and 53.1% (*blue-colored area*) of the total liver volume, respectively

**Fig. 2:**
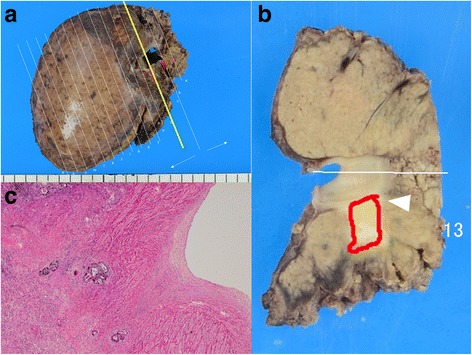
Pathological findings for tumor 1. **a** The resected specimen in segment 8 with the right hepatic vein (RHV). **b** The cut surface of the yellow line in **a**. Tumor 1 (*white arrowhead*) grossly measured 1.0 × 0.6 cm and had invaded the RHV. **c** The histological diagnosis was compatible with metastatic carcinoma of the sigmoid colon, and invasion into the RHV was confirmed

**Fig. 3:**
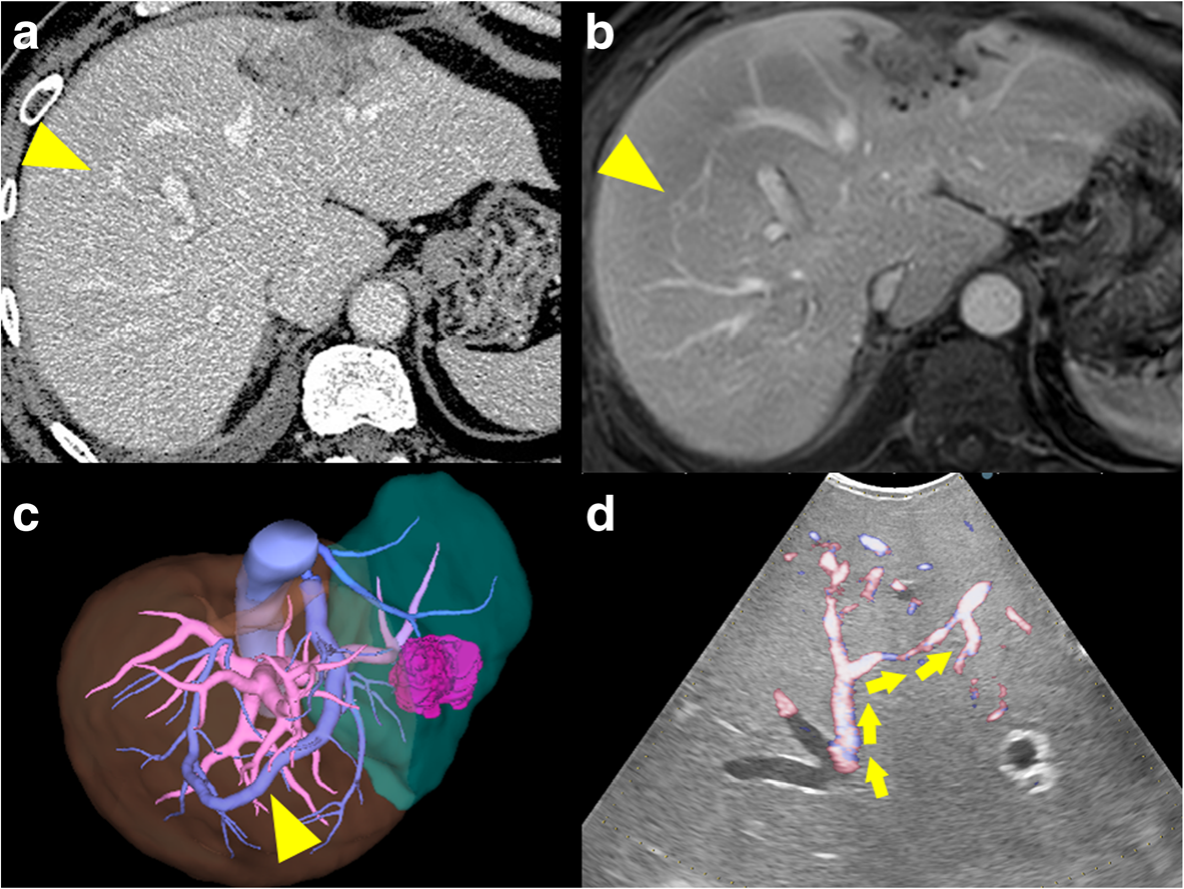
Development of intrahepatic venous collaterals (*yellow arrowhead*) between RHV and MHV after the first hepatectomy. **a** Contrast-enhanced computed tomography. **b** Contrast-enhanced magnetic resonance imaging. **c** Three-dimensional volumetric software. **d** Intraoperative Doppler ultrasonography. Intrahepatic venous collaterals derived from the RHV extended into the MHV (*yellow arrows*)

**Fig. 4:**
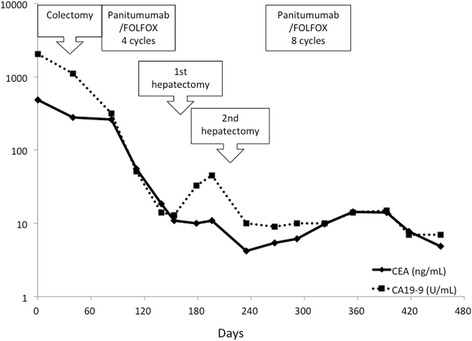
Trends in the tumor markers over the patient’s treatment course. *FOLFOX* 5-fluorouracil, leucovorin, and oxaliplatin, *CEA* serum carcinoembryonic antigen, *CA19-9* serum carbohydrate antigen 19-9

**Fig. 5:**
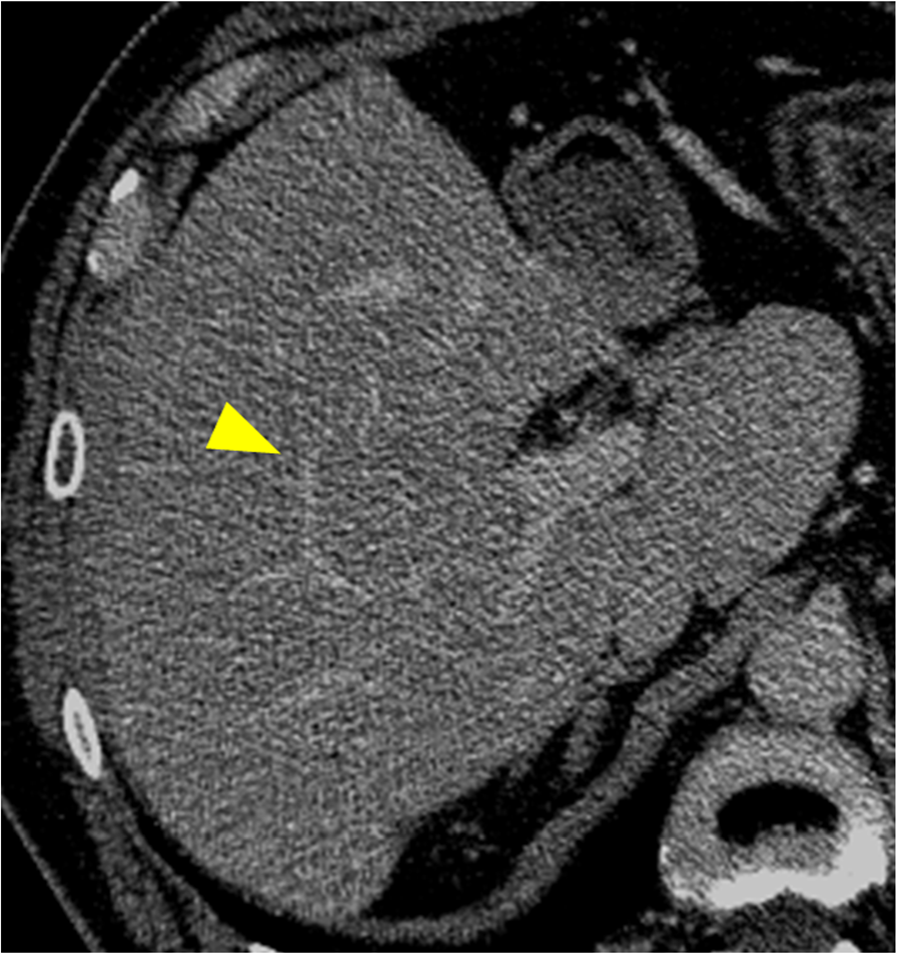
Contrast-enhanced CT performed 10 months after the second hepatectomy. Intrahepatic venous collaterals (*yellow arrowhead*) between the RHV and MHV remained patent

A 60-year-old man was referred to our hospital with the chief complaints of fever and left lower abdominal pain. Serum carcinoembryonic antigen and carbohydrate antigen 19-9 levels were 483.1 ng/ml and 2034 U/ml, respectively. Contrast-enhanced computed tomography (CT) revealed sigmoid colon cancer with intraabdominal abscess and two synchronous liver metastases. One of the liver tumors (tumor 1) was located in segment 8 near the caval confluence. Tumor 1 was 2.2 cm in size and attached to both the right hepatic vein (RHV) and the middle hepatic vein (MHV) (Fig. 1a). The other tumor (tumor 2) was located in the left lobe. Tumor 2 was 10 cm in size and invaded the umbilical portion of the portal vein (Fig. 1b). Sigmoidectomy was performed, and pathological findings revealed moderately differentiated tubular adenocarcinoma. The depth of the tumor was subserosal, and there was no lymph node metastasis. Rat sarcoma viral oncogene homolog was the wild type. Hepatic resection was initially contraindicated because all three major hepatic veins were invaded by the tumors [5]. Tumor 1 near the caval confluence had invaded both the RHV and the MHV, and we could not preserve the left hepatic vein during left hemihepatectomy for tumor 2. Therefore, the patient underwent four cycles of panitumumab/5-fluorouracil, leucovorin, and oxaliplatin (FOLFOX) therapy after sigmoidectomy; following which, both liver metastases decreased in size. Tumor 1 remained attached to the RHV; however, it was located a short distance from the MHV (Fig. 1c). Serum carcinoembryonic antigen and carbohydrate antigen 19-9 levels decreased to 18.7 ng/ml and 14 U/ml, respectively, and the 15-min indocyanine green retention rate was 9.7%. Based on these findings, we planned radical liver resection. Three-dimensional volumetric software (Synapse Vincent^*®*^; Fujifilm, Tokyo, Japan) calculated the total liver volume (TLV) at 1344 ml with estimated volumes for limited resection of segment 8 and left hemihepatectomy of 13 ml (0.9% of TLV) and 263 ml (19.6% of TLV), respectively. When the RHV was severed during limited resection of segment 8, the non-congested FLR volume was estimated at 354 ml (26.3% of TLV) because the congested FLR volume was calculated at 714 ml (53.1% of TLV) (Fig. 1d). We devised a novel TSH procedure because the small non-congested FLR volume was a potential risk for postoperative POLF [3]. We first performed limited resection of segment 8 with resection of the root of the RHV. The left portal vein was neither ligated nor embolized because most of the umbilical portion was invaded by tumor 2 and because we expected not to cause adhesions around the porta hepatis for the second hepatectomy. Macroscopic examination of the resected specimen revealed that the size of tumor 1 was 1.0 × 0.6 cm, and it had invaded the RHV (Fig. 2a, b). Histological diagnosis was compatible with metastatic carcinoma of the sigmoid colon, and invasion of the RHV was confirmed (Fig. 2c). Surgical margins were negative for cancer. The patient was discharged on postoperative day 9 without complications. No local recurrence or new metastatic lesions were detected by contrast-enhanced CT or gadolinium-ethoxybenzyl diethylenetriamine pentaacetic acid-enhanced magnetic resonance imaging performed 38 and 42 days after the first hepatectomy, respectively. CT and magnetic resonance imaging also confirmed the development of intrahepatic venous collaterals between the RHV and MHV (Fig. 3a, b). Three-dimensional volumetric software estimated the non-congested FLR volume at 1242 ml (78.5% of TLV) (Fig. 3c). Both serum aspartate aminotransferase level and alanine aminotransferase level were within normal limits. The 15-min indocyanine green retention rate was considered to keep less than 10% as same as before the first hepatectomy because the patient underwent no chemotherapy during a waiting period; therefore, left hemihepatectomy was performed 58 days after the first hepatectomy. Intraoperatively, we saw no adhesions around the porta hepatis, and the left hepatic artery and the left branch of the portal vein were safely exposed and divided. Intraoperative Doppler ultrasonography confirmed the presence of intrahepatic venous collaterals arising from RHV to MHV (Fig. 3d). The patient’s postoperative course was uneventful, and he was discharged on postoperative day 9 with no signs of POLF. He underwent eight cycles of panitumumab/FOLFOX therapy for 5 months after the second hepatectomy, and his clinical course is summarized in Fig. 4. Contrast-enhanced CT performed 10 months after the second hepatectomy revealed that the intrahepatic venous collaterals remained patent (Fig. 5).

## Discussion

We discussed a novel TSH procedure that allows time for intrahepatic venous collaterals to develop between operations. This strategy is simple and safe and prevents POLF without hepatic venous reconstruction (1) when the tumor invades the root of major hepatic veins, (2) when the majority of the FLR may become congested by severing the root of the hepatic veins, and (3) when the estimated non-congested FLR volume is small following major hepatectomy.

Liver congestion secondary to sacrificing the hepatic veins increases the risk of POLF in liver surgery [3, 4]; however, there are no standard criteria to determine the necessity of hepatic venous reconstruction. Mise et al. reported that the major hepatic veins could be safely sacrificed when the estimated non-congested FLR volume was > 40% of TLV in patients with normal liver function confirmed by a 15-min indocyanine green retention rate < 10% [3]. Based on these criteria, we considered that sacrificing the RHV was unsafe because the estimated non-congested FLR volume in our patient was 26.3% of TLV. Also, in our patient, one-stage hepatectomy, such as reconstruction and partial resection of the RHV [12], was a therapeutic choice. However, one-stage hepatectomies may be appealing, but these procedures are technically challenging, and obstruction of the reconstructed veins can be lethal [9].

In considering how to proceed, we reviewed reports of intrahepatic venous collaterals involving cast studies, Doppler ultrasonography, and CT [11, 13–16]. Hribernik et al. reported that intrahepatic venous anastomoses were found in 46% of normal livers by cast study. In particular, anastomoses between the RHV and MHV were detected in 24% of the livers, and RHV-MHV anastomoses were most commonly seen in segment 8 [11]. Hepatic venous flow can compensate in patients with pre-existing intrahepatic venous anastomoses, intrahepatic collateral development, or reversal of portal flow after hepatic vein ligation [11]. We took advantage of this compensatory ability in the TSH procedure we used in our patient.

The advantages of TSH are that it results in a sufficient FLR and that it allows physicians to select patients eligible for radical surgery because of the wait time between the two operations; however, the development of severe adhesions after the first hepatectomy is a major disadvantage during the second hepatectomy. In our TSH, we performed limited resection of segment 8 first because severe adhesions around the porta hepatis and cut surface of the remnant liver were possible after left hemihepatectomy.

In our patient, RHV-MHV anastomoses were not seen on contrast CT before the first hepatectomy; therefore, the intrahepatic collaterals developed after severing the RHV. Previous reports revealed that these collaterals could develop approximately 10 days after severing the major hepatic veins [17]. Based on this finding, we could have shortened the interval between the two hepatectomies, in our patient.

The limitation of our strategy is that the availability of intrahepatic venous collaterals is uncertain. Several vascular substitutes for hepatic venous reconstruction have been reported including autologous intraabdominal or extraabdominal veins, synthetic grafts such as polytetrafluoroethylene, cryopreserved veins, veins from resected livers, and parietal peritoneum [5–9]. Dokmak et al. summarized the features of these substitutes based on six characteristics: availability, difficulty of harvesting, necessity of additional incision, risk of infection, need for long-term anticoagulation, and cost [8]. Intrahepatic venous collaterals can be an ideal vascular substitute except when considering availability.

## Conclusions

Our TSH procedure contributed to avoiding POLF by allowing time for intrahepatic venous collaterals to develop and did not involve venous reconstruction. This strategy is indicated for patients: (1) with liver tumors invading the root of the major hepatic veins, (2) when the FLR is expected to be markedly congested by severing the root of the hepatic veins, and (3) when the non-congested FLR volume is estimated to be too small to perform major hepatectomy.

## Abbreviations

CT: Computed tomography
FLR: Future liver remnant
FOLFOX: 5-Fluorouracil, leucovorin, and oxaliplatin
MHV: Middle hepatic vein
POLF: Postoperative liver failure
RHV: Right hepatic vein
TLV: Total liver volume
TSH: Two-stage hepatectomy
